# Clinical evaluation of rapid point-of-care antigen tests for diagnosis of SARS-CoV-2 infection

**DOI:** 10.1007/s10096-021-04274-7

**Published:** 2021-05-22

**Authors:** Johannes G. M. Koeleman, Henk Brand, Stijn J. de Man, David S. Y. Ong

**Affiliations:** 1 Department of Medical Microbiology and Infection Control, Franciscus Gasthuis & Vlietland, Kleiweg 500, 3045 PM Rotterdam, The Netherlands; 2 Department of Epidemiology, Julius Center for Health Sciences and Primary Care, University Medical Center Utrecht, Utrecht, The Netherlands; 3 Present Address: Rotterdam, The Netherlands

**Keywords:** SARS-CoV-2, COVID-19, Rapid antigen test, Lateral flow immunoassay, POC test

## Abstract

The RT-qPCR in respiratory specimens is the gold standard for diagnosing acute COVID-19 infections. However, this test takes considerable time before test results become available, thereby delaying patients from being diagnosed, treated, and isolated immediately. Rapid antigen tests could overcome this problem. In the first study, clinical performances of five rapid antigen tests were compared to RT-qPCR in upper respiratory specimens from 40 patients with positive and 40 with negative RTq-PCR results. In the second study, the rapid antigen test with one of the best test characteristics (Romed) was evaluated in a large prospective collection of upper respiratory specimens from 900 different COVID-19-suspected patients (300 emergency room patients, 300 nursing home patients, and 300 health care workers). Test specificities ranged from 87.5 to 100.0%, and test sensitivities from 55.0 to 80.0%. The clinical specificity of the Romed test was 99.8% (95% *CI* 98.9–100). Overall clinical sensitivity in the study population was 73.3% (95% *CI* 67.9–78.2), whereas sensitivity in the different patient groups varied from 65.3 to 86.7%. Sensitivity was 83.0 to 86.7% in patients with short duration of symptoms. In a population with a COVID-19 prevalence of 1%, the negative predictive value in all patients was 99.7%. There is a large variability in diagnostic performance between rapid antigen tests. The Romed rapid antigen test showed a good clinical performance in patients with high viral loads (RT-qPCR cycle threshold ≤30), which makes this antigen test suitable for rapid identification of COVID-19-infected health care workers and patients.

## Introduction

Accurate and early diagnosis of severe acute respiratory syndrome coronavirus 2 (SARS-CoV-2) infection is crucial for patient management and outbreak control of the coronavirus disease 2019 (COVID-19) pandemic. The quantitative reverse transcription polymerase chain reaction (RT-qPCR) assay for the detection of SARS-CoV-2 virus in respiratory specimens still remains the gold standard for diagnosing COVID-19 [1]. A major drawback of this sensitive and specific molecular diagnostic method is the limited worldwide availability in combination with a relatively long turnaround time.

The development of rapid diagnostic tests allows faster identification of COVID-19 patients and enables the prompt implementation of infection prevention and control measures. Therefore, a large number of COVID-19 point-of-care (POC) antigen tests with rapid turnaround time and minimal need for instrumentation have been developed and introduced recently [2–7]. These easy to perform and inexpensive POC tests based on lateral flow immunochromatographic assays (LFAs) can detect nucleocapsid protein from SARS-CoV-2 in nasopharyngeal specimens within 20 min, which makes them ideal for use in patient care settings but also in the community [8].

Until now, little is known about the SARS-CoV-2 antigen test performance in different patient groups. In this study, we evaluated the clinical performance of different LFAs compared to RT-qPCR using upper respiratory specimens from several patient groups with suspected COVID-19.

## Materials and methods

### Study design

For both studies described here, a prospective collection of upper respiratory specimens sent to the microbiology laboratory was used. In the periods from October 6 to October 12 (study 1) and October 24 to November 15, 2020 (study 2), samples were obtained from different COVID-19-suspected emergency room patients (ERP), nursing home patients (NHP), and health care workers (HCW) with acute onset of one or more respiratory symptoms (cough, sore throat, dyspnea, coryza, anosmia, ageusia) with or without fever. During the 19-day study period, the mean proportion of newly confirmed RT-qPCR-positive COVID-19 cases per day was 23%. The study was conducted in a teaching hospital (Franciscus Gasthuis & Vlietland, Rotterdam, the Netherlands).

### Sample collection and storage

Patients and HCW with suspected COVID-19 infection were sampled by one combined throat nasopharyngeal swab. After this, swabs were placed in 3 ml virus transport medium (VTM) and stored at 4°C until sample preparation after which positive samples were stored at −20°C. All specimens were examined for SARS-CoV-2 viral RNA by routine RT-qPCR on the day of collection and for antigen detection by three of five LFAs within 72 h and by two LFAs 1 month after collection.

### Study 1 (Comparison of five rapid antigen tests)

The first study involved a comparison of five different COVID-19 rapid antigen tests for the detection of SARS-CoV-2 viral antigens. From October 6 to October 12, 2020, consecutive patient samples were included for a total of 40 RT-qPCR-negative and 40 RT-qPCR-positive samples with a proportional number of PCR-positive samples divided into different cycle threshold (C_*T*_) categories (C_*T*_ < 20, C_*T*_ 20–30 and C_*T*_ > 30). In the first part, three LFAs available at that time were evaluated: Certest SARS-CoV-2 (Certest Biotec S.L., Spain), Roche SARS-CoV-2 Rapid Antigen Test (Roche, Switzerland), and Romed Coronavirus Ag Rapid Test (Romed, The Netherlands). In the second part, the Romed Coronavirus Ag Rapid Test and two WHO-recommended LFAs, i.e., BD Veritor SARS-CoV-2 point-of-care test (Becton, Dickinson and Company, USA) and Panbio™ COVID-19 Antigen rapid test (Abbott, USA), were evaluated with 40 RT-qPCR-positive samples: 35 samples that were stored at −20°C from the first part, completed with five additional RT-qPCR-positive samples with corresponding C_*T*_ values of the missing samples. The BD Veritor and Panbio™ LFAs were not available initially and were therefore added in the separate second comparison at a later moment.

### Study 2 (Romed-RT-qPCR comparison)

In the second prospective study, the clinical performance of the Romed LFA was compared to RT-qPCR in an extended cohort of patients and HCW of which consecutive samples were included between October 24 and November 15, 2020. During the inclusion, patient categories (ERP, NHP, or HCW) and PCR results (positive and negative) were recorded. Inclusion of new samples in a particular patient category was stopped after a predefined number of 300 PCR-positive and 600 PCR-negative samples had been included divided among the three defined patient groups.

### Detection of viral RNA by direct RT-qPCR methods

Samples from NHP and HCW were tested on two different RT-qPCR methods by either a validated in-house RT-qPCR assay or on the ELITe InGenius® (Elitech, France) platform [9]. Samples from ERP were tested with the GeneXpert Xpress SARS-CoV-2 PCR assay (Cepheid Inc., Sunnyvale, USA) according to the instructions of the manufacturer.

### Detection of SARS-CoV-2 viral antigen by LFAs

For antigen extraction, 350 μl of VTM was added to 300 μl of each respective extraction buffer and mixed for 10 s. Subsequently, a number of drops of the mixture were added to the sample port of the antigen assay according to the instructions of the manufacturer. The result was read visually after 15 min whereby any shade of color in the test line region was considered positive. All tests were independently assessed by two investigators who were blinded to all other test results, and in case of discrepancy, an additional assessment was performed by a third investigator.

### Ethical statement

The Institutional Review Board waived the need for informed consent because tests were performed on samples that had been required for routine microbiological investigation (IRB protocol number 2020-109). Also according to hospital procedure, all patients were informed about the possibility of an opt-out if they had objections against the use of leftover material for research to improve or validate diagnostic testing procedures. The study was performed in accordance with Helsinki Declaration as revised in 2013.

### Data collection and statistical analysis

The primary outcome measures for both studies were clinical specificity and clinical sensitivity in relation to different C_*T*_ values of the RT-qPCR. For the second study, positive predictive value (PPV) and negative predictive value (NPV) were also calculated as secondary outcomes in order to develop a diagnostic algorithm in different patient groups. All data were analyzed using Microsoft Excel, GraphPad Prism version 8, and R version 3.3.2 (R Foundation for Statistical Computing). Groups were compared by using Mann-Whitney U test for continuous variables and chi-square test or Fisher’s exact test for categorical variables as appropriate. Values of p that were < 0.05 were considered to be statistically significant.

## Results

### Study 1 (Comparison of five rapid antigen tests)

In part one of this study, three COVID-19 rapid antigen tests were compared to RT-qPCR in 80 selected specimens of which 40 were RT-qPCR negative and 40 were positive with different viral loads. The performance of the LFAs varied greatly as shown in Table 1, with an overall sensitivity ranging from 55.0% (95% confidence interval (CI) 38.7–70.4) (Certest) to 72.5% (95% CI 55.9–84.9) (Romed). In specimens with a high viral load (C_*T*_ 30 or lower), sensitivity of all the assays increased. The specificity was 87.5% or higher for all LFAs, and the Romed test showed 100% specificity.

**Table 1:** Performance characteristics of five COVID-19 rapid antigen tests on throat and nasopharyngeal samples compared to RT-qPCR

Assay	First part^a^			Second part^b^		
	Romed	Roche	Certest	Romed	Panbio	BD Veritor
Specificity % (95% *CI*)	100 (89.0–100)	87.5 (72.4–95.3)	97.5 (85.3–99.9)	ND	ND	ND
Sensitivity % (95% *CI*)						
Overall	72.5 (55.9–84.9)	62.5 (45.8–76.8)	55.0 (38.7–70.4)	80.0 (63.8–90.4)	70.0 (53.3–82.9)	77.5 (61.1–88.6)
C_T_ < 30	93.3 (76.5–98.8)	83.3 (64.5–93.7)	73.3 (53.8–87.0)	96.7 (80.9–99.8)	86.7 (68.4–95.6)	93.3 (76.5–98.8)
C_T_ < 20	100 (65.5–100)	100 (65.5–100)	100 (65.5–100)	100 (65.5–100)	100 (65.5–100)	100 (65.5–100)
True positives	29	25	22	32	28	31
False positives	0	5	1			
False negatives	11	15	18	8	12	9
True negatives	40	35	39			

^a^Assays were tested with 40 RT-qPCR-positive and 40 RT-qPCR-negative samples

^b^Assays were tested with 35 RT-qPCR-positive samples from part one supplemented with 5 other RT-qPCR-positive samples. Specificity, sensitivity, PPV, and NPV are reported with 95% *CI*

*CI* confidence interval, *ND* not determined, *C*_*T*_ cycle threshold, *PPV* positive predictive value, *NPV* negative predictive value

Clinical sensitivity of the BD Veritor and the Panbio™ LFAs was 77.5% (95% *CI* 61.1–88.6) and 70.0% (95% *CI* 53.3–82.9), respectively. In order to compare the clinical sensitivity of all the LFAs tested, the Romed antigen test was also performed with these samples. The sensitivity of the Romed in the second comparison increased to 80.0% (95% *CI* 63.8–90.4) indicating that storage of the samples did not affect LFA results and thus allows a sensitivity comparison of all LFAs tested. From all the five LFAs tested, the Romed LFA showed the highest clinical sensitivity. Based on these results, we decided to continue with the Romed antigen test in the second study as it had one of the best test characteristics.

### Study 2 (Romed-RT-qPCR comparison)

A total of 900 throat nasopharyngeal swabs (300 in each patient category: ERP, NHP, and HCW) were prospectively selected. This resulted in 300 (33.3%) samples that were tested positive for SARS-CoV-2 by RT-qPCR. Compared to RT-qPCR, the clinical specificity of the Romed test was 99.8% (95% *CI* 98.9–100) (Table 2). Only one false-positive LFA result was found in a swab from an HCW. Discrepancies in test results between different investigators were seen in only 7 of the 900 (0.8%) antigen tests performed. Overall clinical sensitivity in the study population was 73.3% (95% *CI* 67.9–78.2). Sensitivity in the different groups varied from 65.3% (95% *CI* 57.1–72.8) for ERP, 76.0% (95% *CI* 64.5–84.8) for NHP to 86.7% (95% *CI* 76.4–93.1) for HCW. PPV for all patients were 81.6% (95% *CI* 38.5–96.9) and 99.3% (95% *CI* 95.4–99.9) at a prevalence of 1% and 25%, respectively. In contrast, the NPV for the ERP, NHP, and HCW in a population prevalence of 1% was at least 99.7%, whereas at a population prevalence of 25%, these were 89.6% (95% *CI* 87.4–91.5), 92.6% (95% *CI* 89.3–94.9), and 95.7% (95% *CI* 92.6–97.6), respectively.

**Table 2:** Results of Romed COVID-19 antigen test compared to RT-qPCR on 900 throat nasopharyngeal samples

Patients	Emergency room patients	Nursing home residents	Health care workers	All patients
RT-qPCR-negative samples	150	225	225	600
RT-qPCR-positive samples	150	75	75	300
Specificity (95% *CI*)	100 (96.9–100)	100 (97.9–100)	99.6 (97.2–100)	99.8 (98.9–100)
Sensitivity (95% *CI*)				
Overall	65.3 (57.1–72.8)	76.0 (64.5–84.8)	86.7 (76.4–93.1)	73.3 (67.9–78.2)
C_*T*_ *< 30*	90.6 (82.9–95.1)	85.1 (73.8–92.2)	92.6 (83.0–97.3)	89.6 (84.9–93.0)
C_*T*_ < 20	100 (84.0–100)	100 (82.2–100)	100 (79.1–100)	100 (99.3–100)
PPV (95% *CI*)				
Prevalence 1%	100 (N.A.)	100 (N.A.)	66.3 (22.8–93.3)	81.6 (38.5–96.9)
Prevalence 10%	100 (N.A.)	100 (N.A.)	95.6 (75.4–99.4)	98.0 (87.3–99.7)
Prevalence 25%	100 (N.A.)	100 (N.A.)	98.5 (90.2–99.8)	99.3 (95.4–99.9)
Prevalence 50%	100 (N.A.)	100 (N.A.)	99.5 (96.5–99.9)	99.8 (98.4–100)
NPV (95% *CI*)				
Prevalence 1%	99.7 (99.6–99.7)	99.8 (99.6–99.8)	99.9 (99.8–99.9)	99.7 (99.7–99.9)
Prevalence 10%	96.3 (95.4–97.0)	97.4 (96.2–98.3)	98.5 (97.4–99.2)	97.1 (96.5–97.6)
Prevalence 25%	89.6 (87.4–91.5)	92.6 (89.3–94.9)	95.7 (92.6–97.6)	91.8 (90.3–93.1)
Prevalence 50%	74.3 (69.8–78.2)	80.7 (73.6–86.2)	88.2 (89.6–95.7)	78.9 (75.6–81.9)

Specificity, sensitivity, PPV, and NPV are reported with 95% CI. The PPV was calculated for 4 scenarios: 1% and 10% prevalence in a general population and 25 and 50% prevalence in a high-risk population

*CI* confidence interval, *C*_*T*_ cycle threshold, *N.A.* not applicable

**Fig. 1:**
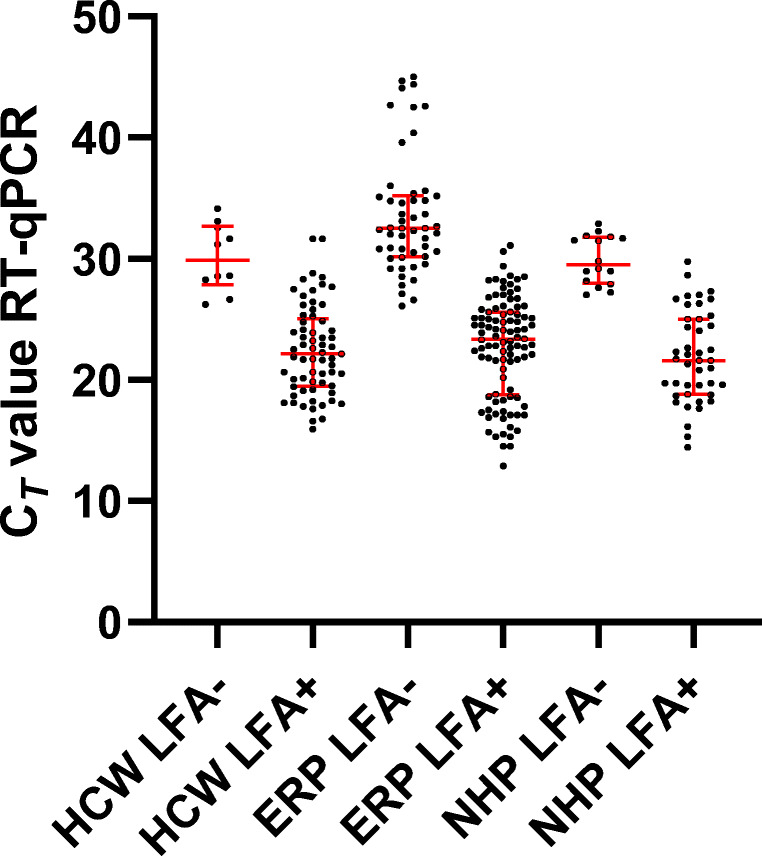
Correlations of cycle threshold (C_*T*_) values of RT-qPCR E gene and the LFA rapid antigen results of throat nasopharyngeal samples with positive and negative results from 225 health care workers (HCW), 150 emergency room patients (ERP), and 225 nursing home residents (NHP). The median C_*T*_ values and interquartile range are shown in red

The median C_*T*_ value for E gene of all 300 RT-qPCR-positive patients was 25 (interquartile range (*IQR*) 21–29). The median C_*T*_ E gene value of LFA-positive patients was 23 (*IQR* 19–25) compared to 32 (*IQR* 29-34) of LFA-negative patients (p < 0.01), and C_*T*_ values were also statistically significantly different between LFA-positive and LFA-negative cases in each subgroup (all p < 0.01) (Fig. 1). In the group of ERP, the sensitivity of the antigen test on RT-qPCR-positive samples with a C_*T*_ value lower than 30 was 90.6% (95% *CI* 82.9–95.1). Clinical sensitivity of 100% was found in all patient categories with C_*T*_ values below 20 which corresponds with high viral loads.

**Fig. 2:**
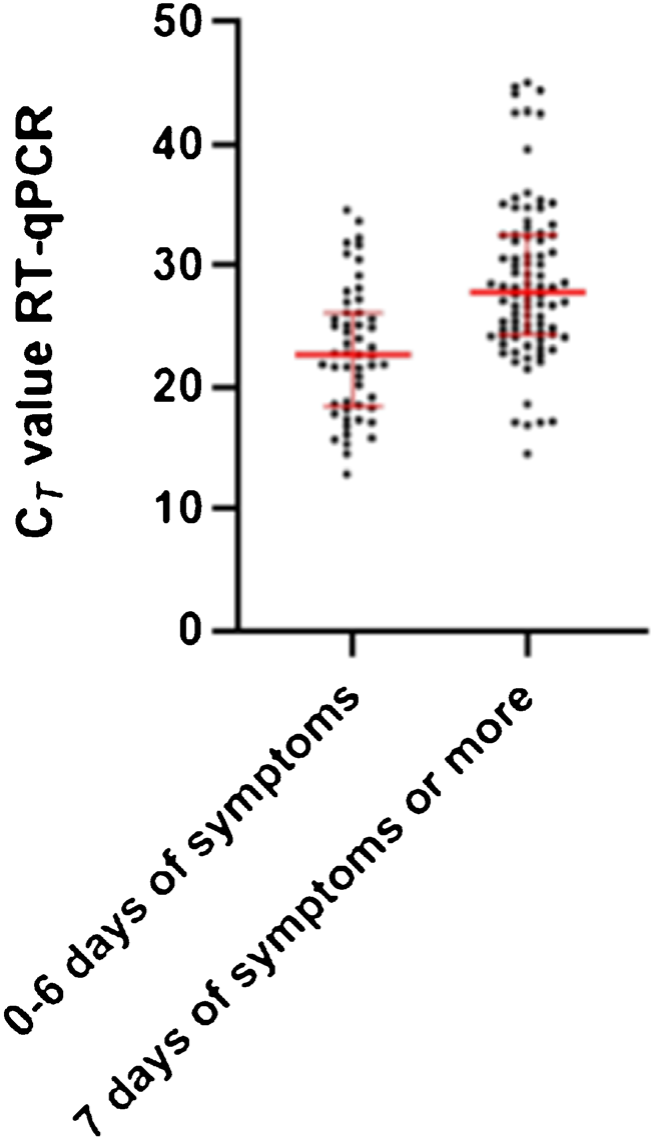
Distribution of cycle threshold (C_*T*_) values of RT-qPCR E genes and duration of symptoms in 150 emergency room patients (ERP). The median C_*T*_ values and interquartile range are shown in red

**Fig. 3:**
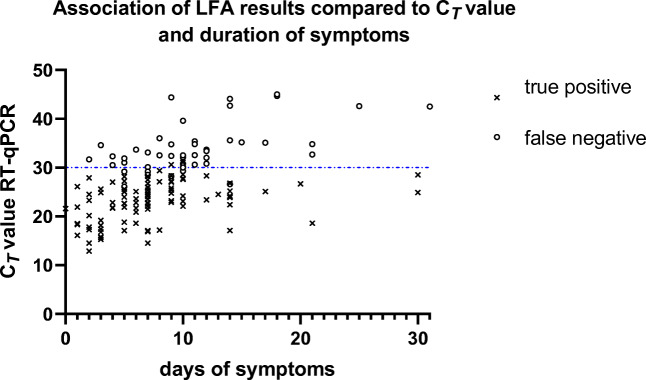
Association between LFA results (Romed), C_*T*_ values of RT-qPCR-positive throat nasopharyngeal samples, and duration of symptoms in patients presenting to the emergency department

The LFA results in ERP and HCW in this study showed a high sensitivity in samples obtained during the first week of symptoms. This was seen in HCW of whom the majority was tested within the first week after symptom onset and a clinical sensitivity of 86.7% was found. For ERP with symptoms less than 7 days and 7 days or more since onset, the sensitivity was 83.0% (95% *CI* 69.7–91.5) and 56.2% (95% *CI* 45.3–66.5) (p < 0.01), respectively, with significant lower C_*T*_ values in the first group (p < 0.01) (Fig. 2). Also, in this group, false-negative LFA results were only seen in RT-qPCR-positive samples with high *C*_*T*_ values (Fig. 3).

## Discussion

In this study, comparison of five different LFAs performed on patients suspected for COVID-19 showed a very high specificity and a moderate to high sensitivity ranging from 55 to 80%. Awareness of the lack of sensitivity in rapid antigen tests is important especially considering the criteria established by the European Centre for Disease Prevention and Control and the World Health Organization [10]. Sensitivities of these LFAs increased above 90% when considering specimens with C_*T*_ values below 30 (i.e., higher viral loads), for the two best performing antigen tests. Compared to RT-qPCR, samples from the upper respiratory tract (nasal or nasopharyngeal swabs) have shown a highly variable sensitivity, ranging from 0 to 94% but with a high reported specificity (i.e., more than 97%) [7]. Another recent study showed differences in sensitivity of commercially available rapid antigen LFAs, which correlates with the ability to detect infectious COVID-19 patients [11]. In our study, we also observed a wide variation in the sensitivity of the five LFAs tested with a high sensitivity found for the Romed antigen test.

In our second study, the clinical performance of the Romed LFA antigen test was evaluated in 900 patients showing an overall specificity and sensitivity of 99.8% and 73.3% respectively, compared to RT-qPCR. This is the first study in which the clinical performance of the Romed LFA in a large proportion of samples collected from different patients has been evaluated. Our study shows that the sensitivity of this LFA test was moderate in patients suspected for COVID-19 presenting to the hospital, but increased in patients with symptoms for less than a week and also in HCW with short duration of symptoms. This is in accordance with the higher sensitivity found in samples with a low C_*T*_ value corresponding to infected patients with a high viral load. Recently, two other reports have evaluated the performance of a rapid antigen test for COVID-19 community screening in individuals with COVID-19-like symptoms [12, 13]. In these studies, specificities of 100% were found whereas sensitivities of two studies with the Panbio™ test were 72.6% and 81.0%, and of the one with the BD Veritor 80.7%. Another study in 150 emergency room patients and 105 from primary health care centers sampled during the first week of symptoms showed a Panbio™ sensitivity of 73% [14]. These results are comparable with the sensitivities in HCW (86.7%) and NHP (76.0%) found in our study both reflecting individuals and patients with a recent COVID-19 infection. The majority of false-negative results were found in samples with high C_*T*_ levels corresponding with low viral loads and longer durations of symptoms. In addition, several studies have shown a clear association between high C_*T*_ values of RT-qPCR (i.e., above 30) and low COVID-19 patient infectivity [15, 16]. Therefore, the Romed LFA could be used in admitted COVID-19 patients in order to determine their infectivity which offers the opportunity for earlier discontinuation of isolation.

The clinical performance of COVID-19 rapid antigen tests largely depends on the prevalence of COVID-19 as well as the different patient populations in which they are used. The negative predictive value increases when the prevalence decreases. In settings where the pre-test probability of having COVID-19 is low and symptom duration is short, a negative LFA antigen result could exclude that person from the need of further testing, whereas positive LFA antigen results need to be confirmed by RT-qPCR because the PPV is only moderate when the prevalence is low and it may result in substantial false-positives. According to our findings, this is especially the case for HCW in a low prevalence setting (Table 2). Based on our results, the application of Romed LFAs in HCW with short duration of symptoms can reliably identify COVID-19-positive contagious individuals. In contrast, in ERP with a high pre-test probability of having COVID-19, more false-negative results were observed which makes the use of these LFAs in this setting less suitable. Finally, in addition to RT-qPCR testing, Romed LFAs could be used for frequent and repeat screening of nursing home residents in outbreak situations for rapid identification and isolation of COVID-19 patients in order to prevent further transmission at an early stage.

Our study has several limitations. First, all LFAs were performed on VTM and not on the originally collected throat nasopharyngeal swabs in order not to affect the routine COVID-19 diagnostics by RT-qPCR and to be able to compare 5 different LFAs head-to-head. This could theoretically have influenced test characteristics; however, the results of our study are in line with the results of other studies published [12–14]. Second, due to the late availability of two of the five LFAs tested, positive samples were frozen and used after thawing which also is not according to the manufacturers’ instructions. Therefore, the Romed LFA was included in both the first and the second LFAs antigen comparison. Importantly, we did not observe a decrease in sensitivity when tested after freezing and thawing. Third, samples used in the second study were partially selected (i.e., in favor of RT-qPCR-positive samples) in order to obtain a high number of positive samples to allow adequate assessments of sensitivity characteristics in the different patient groups.

In our study, the Romed LFAs have a high sensitivity and specificity in throat nasopharyngeal samples with high viral loads which make them most suitable for rapid identification and isolation of COVID-19-infected and contagious HCW and patients. The worldwide spread of SARS-CoV-2 has resulted in a large number of COVID-19 patients. In order to reduce or prevent further spread in health care facilities and in the community, quick and accurate identification of COVID-19 patients followed by quarantine measures is essential. Therefore, in addition to RT-qPCR, COVID-19 rapid antigen tests, which are simple, rapid, inexpensive, and appropriate for wide-scale use, offer the opportunity to help with this containment strategy.
